# Paraganglioma of the Seminal Vesicle Case Report and Review of the Literature

**DOI:** 10.1089/cren.2016.0119

**Published:** 2016-12-01

**Authors:** Georgi Tosev, Michael Mendler, Frank Bergmann, Tilman Klein, Sascha A. Pahernik, Boris A. Hadaschik, Markus Hohenfellner

**Affiliations:** ^1^ Department of Urology, University of Heidelberg, Heidelberg, Germany.; ^2^ Department of Medicine 1 and Clinical Chemistry, University of Heidelberg, Heidelberg, Germany.; ^3^ Department of Pathology, University of Heidelberg, Heidelberg, Germany.

**Keywords:** DaVinci robot, ectopic paraganglioma, seminal vesicle

## Abstract

***Background:*** The present case report describes an extremely rare case of a norepinephrine secreting extraadrenal paraganglioma (PGL) located in the seminal vesicle.

***Case Presentation:*** A 36-year-old patient had signs of intermittent hypertensive derailments, bradycardia, increased norepinephrine excretion in 24-hour urine, an increased metanephrine plasma concentration, and a positive clonidine suppression test. A suspicious mass was detected in an (18)F-DOPA-PET/CT-scan in the left seminal vesicle. Following adrenergic blockade, a robotically assisted laparoscopic left vesiculectomy with negative soft tissue surgical margins was performed. The patient sustained a couple of few months of voiding difficulties of the lower urinary tract and obstruction of the left upper urinary tract after the surgery, which resolved spontaneously with home medical treatment. Two years after the initial treatment, the patient relapsed, which was confirmed by endocrinologic follow-up tests with increased urine catecholamine, a positive clonidine suppression test, as well as an elevated blood pressure. Staging with (18)F-DOPA-PET/CT-scan confirmed the diagnosis of a recurrent PGL. This was followed by subsequent open surgical removal of the suspicious lesion in the seminal fossa. The patient is still recurrence free 15 months after the second surgery. Complications after the second surgery included an intermittent paresthesia of the left leg lasting 3 to 4 months. No other urologic symptoms such as voiding or erectile dysfunction occurred.

***Conclusion:*** DaVinci-assisted laparoscopic vesiculectomy is a viable procedure to treat such cases providing satisfactory results. Relevant for clinical practice are the regular and lifelong follow-up examinations to detect recurrences.

## Introduction and Background

Paragangliomas (PGL) are extraadrenal neuroendocrine tumors that arise from an autonomous ganglion (paraganglion) and may occur at various body sites. They originate from chromaffin cells in the paraganglia in localizations such as the retroperitoneum and thorax in the sympathetic system or they can be of parasympathetic origin and are localized, for example, in the aortic arch, neck, and skull base.^1^

PGL may produce and release surplus catecholamines (mostly normetanephrine and norepinephrine) into the blood. This leads to intermittent or permanent hypertension, episodic sweating, headaches, and palpitations.^2^ Most PGL are benign tumors, but depending on the location and presence of genetic mutations, the probability of malignant PGL reaches up to 25% metastatic potential. The highest rate of metastases occurs in hereditary PGL syndromes and is associated with mutations of genes of the succinate dehydrogenase complex (SDH gene). The majority of PGL-related metastases occur in local and distant lymphatic nodes, bones, liver, and lungs.

Optimal treatment of regional PGL that have spread to neighboring organs or lymph nodes consists of complete surgical removal of the tumor.^2–4^

A localization of a PGL in small pelvis in male is extremely rare. Moreover, so far, only three cases have been described involving the seminal vesicle.^5–7^

## Case Presentation

A 36-year-old patient presented initially with intermittent hypertensive derailments (RR 220/180 mm Hg) of a known arterial hypertension, accompanied by low heart rates of 40/minute at the Heidelberg University Hospital's Endocrinology Department in January 2013 for further diagnostic procedures.

Arterial hypertension was initially diagnosed in 2009. Since then, recurrent hypertensive crises had occurred. The hypertensive episodes were accompanied by weakness, paleness of the face, serious holocephalic headache, nausea, and hyperhidrosis.

The thyroid metabolism in terms of TSH and fT3 values was normal. Also, all the tumor markers such as the carcinoembryonic antigen, the prostate-specific antigen (PSA), and the calcitonin levels were within the normal range. Urologic symptoms such as voiding difficulties, urinary incontinence, erectile dysfunction, or other symptoms involving the lower or upper urinary tract were not present. Physical examination showed no abnormalities. Renal retention parameters were unremarkable. However, an increased noradrenaline excretion in 24-hour urine and an increased metanephrine level in plasma were determined. A clonidine suppression test was carried out in January 2013 and showed an insufficient suppression suspicious of PGL with DD pheochromocytoma (PHEO) of norepinephrine phenotype. However, an external CT scan of the abdomen did not show pathologic changes and in particular no suspicious adrenal masses.

**FIG. 1.**
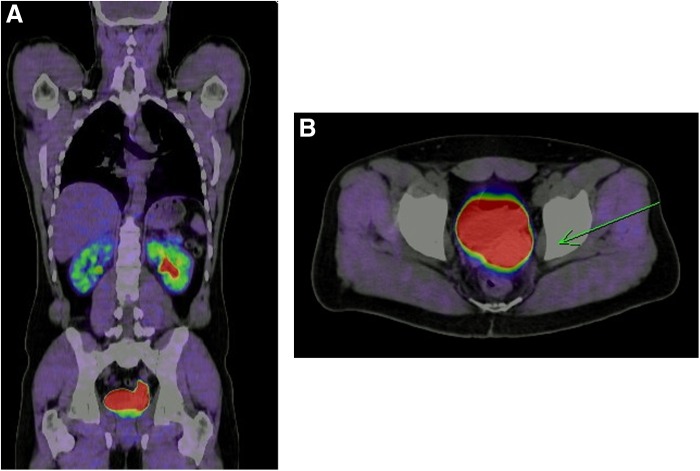
**(A)** Sagittal plane (18)F-DOPA-PET/CT of norepinephrin-producing tumor localized in the left seminal vesicle. **(B)** Transversal plane (18)F-DOPA-PET/CT of norepinephrin-producing tumor localized in the left seminal vesicle.

For further localization of the assumed lesion, a 131IMIBG scintigraphy was performed in February 2013, without clear evidence of a neoplastic lesion. The case was presented in the interdisciplinary tumor conference. For further diagnostics, a CT and (18)F-DOPA-PET/CT examination were performed, which detected a suspicious lesion measuring 3.6 × 2.8 cm in the left seminal vesicle (depicted in Fig. 1A, B). In addition, the staging showed asymptomatic first-grade upper urinary obstruction of the left urinary tract and a primary unspecific nodule with a diameter of 7 mm in segment 8 of the right lung.

## Initial Treatment, Histologic Finding

The patient was initially treated by preoperative alpha- and beta-blockade. Then, a DaVinci-assisted resection of the left tumor-bearing seminal vesicle, using the same approach as the initial step of Monsuru technique for laparoscopic DaVinci-assisted radical prostatectomy, was performed in March 2013.

**FIG. 2.**
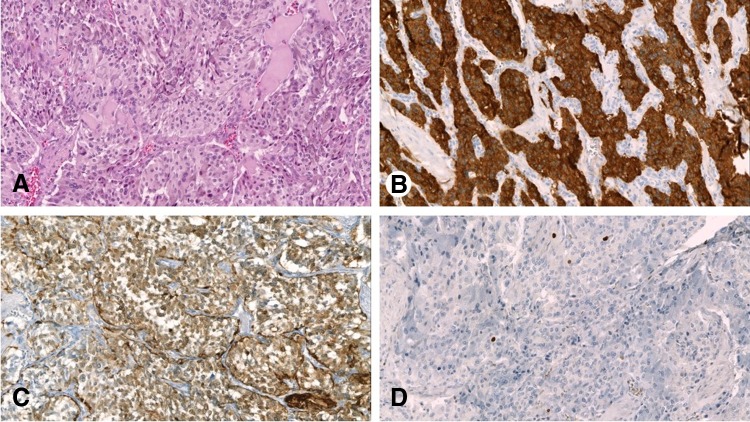
**(A)** Histological aspect of tumor with confluent nests of polygonal tumor cells. **(B)** Immunohistochemical positivity for synaptophysin. **(C)** S100-protein staining revealing few sustentacular cells. **(D)** KI-67 staining with low proliferation activity.

Macroscopic and histopathologic evaluation findings revealed an infiltrative PGL of the seminal vesicle with tumor-free surgical margins (R0) and a maximal width of 3.5 cm. The tumor was composed of confluent nests of moderately polymorphous, polygonal tumor cells (depicted in Fig. 2). One mitosis was detected in 30 high-power fields. Immunohistochemical studies showed a strong expression of chromogranin A, synaptophysin, and neurogenic-specific enolase. In the S-100 staining, few sustentacular cells were observed. The Ki67 proliferation activity (MIB1) of the tumor was low at ∼2%. The tumor cells did not express CK7, CK18, CK20, CDX-2, calretinin, or TTF-1. In the given clinical context, the conventional light microscopic findings, and the immunohistochemical results, the diagnosis of a PGL of the left seminal vesicle was established.

## Postoperative Course

The patient recovered quickly after the procedure. Hypertensive crises and accompanying autonomic symptoms such as headaches, palpitations, sweating, tremor, pallor, or flushing were no longer present after surgery. Plasma normetanephrine and urine metanephrine were normalized after surgery. A second-degree hydronephrosis of the left kidney and high residual volume after voiding >120 mL were observed as temporary postoperative complications. Cystoscopy with retrograde pyelography was performed, in which the prostate was not obstructive but a short distal internal stricture with ∼3 mm length of the left ureter was revealed. A suprapubic bladder catheter and a ureteral stent into the left distal ureter were inserted. After 4 weeks, the ureteral stent was removed and the retrograde pyelogram showed complete drainage of the contrast agent from the left renal pelvis. One follow-up examination with renal function scintigraphy in August 2013 showed no evidence of obstruction or impaired kidney function.

Due to persistent lower urinary tract symptoms (LUTS), the patient wrote a voiding diary in June 2013. After exclusion of mechanical obstructions of a lower urinary tract, a urodynamic examination was performed showing a normal and stable bladder function, normal capacity of the bladder, and a normal detrusor pressure build-up during voiding without any evidence of neurogenic disorder. To stimulate the bladder function, postoperative medical therapy with pyridostigmine (cholinesterase inhibitor) and tamsulosin (selective alpha-1 receptor blocker) was initiated, which resulted in a normalization of the micturition.

Moreover, the suprapubic catheter was removed 4 months postoperatively after improvement of the residual urinary amounts and urine flow. After the surgery, the potency was preserved.

## Follow-Up Seminal Vesiculectomy

The initial follow-up was undertaken ex domo in March 2015.

**FIG. 3.**
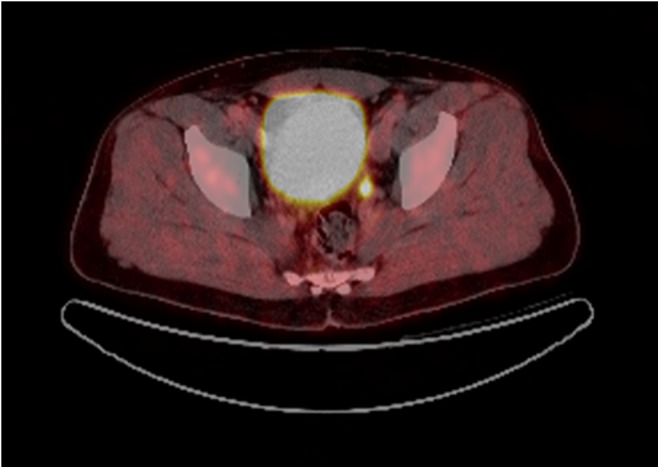
Transversal plane (18)F-DOPA-PET/CT of norepinephrine-producing tumor recurrence localized in the left seminal vesicle.

The endocrinologic tests identified an increased metanephrine level in 24-hour urine, slightly elevated blood pressure, and showed a positive result during the clonidine suppression test. A repeat (18)F-DOPA-PET/CT imaging (depicted in Fig. 3) examination was carried out in March 2015 for staging. PET imaging detected a local recurrence in the left seminal vesicle fossa with dimensions of 0.5 × 2.3 cm. The previously known lesion in the lung had not changed since December 2012.

According to another external multidisciplinary tumor board decision, open surgery via median hypogastric laparotomy incision was performed in May 2015. The new suspect tumor mass in the left seminal fossa was effectively removed. The histopathologic result showed an ∼8 mm measuring PGL. After the second surgery, a sensory disturbance of the left leg persisted for about 12 weeks. Neither a voiding dysfunction with elevated residual urine nor obstructions of the left upper urinary tract occurred. The sexual function was preserved and is still normal postoperatively.

Until now, more than 15 months after recurrence diagnosis and effective surgical treatment, the patient has no complaints and no recurrence of the tumor.

## Discussion

We describe a rare case of symptomatic ectopic PGL of the left seminal vesicle with sympathetic origin and pronounced symptoms of catecholamine excess.

PGL in the seminal vesicle are extremely rare and so far only three cases have been described.^5–7^

The clinical presentation and symptoms: one case presented with signs of acute appendicitis,^7^ one case presented with history of chromophobe renal-cell carcinoma and during a routine follow-up evaluation revealed tumor in the seminal vesicle,^6^ PSA level was measured only in our case and was normal, upper urinary tract obstruction (UUTO) was present in the current case, no signs of hematuria, hematospermia, metastatic disease, or genetic mutation were presented. LUTS were at the present case and for the other three cases were not present.^5–7^ PGL can be divided into functional and nonfunctional types due to their ability for synthesis, storage, and secretion of catecholamine.^2^ In the current case, the patient presented with hypertension due to catecholamine excess.

The primary treatment of extraadrenal PGL consists of surgical resection of the tumor after appropriate pretreatment with alpha- and beta-blockers to block the catecholamine receptors and prevent sympathetic overload and perioperative cardiovascular complications. This is the first published case of a DaVinci-assisted minimally invasive surgery to remove a tumor mass from the area of the seminal vesicle. As for tumor recurrence, after initially negative soft tissue margins, it cannot be clearly established whether it is residual tissue after primary surgery or a second separate tumor.

Advantages of a robotically assisted laparoscopic retroperitoneal approach are minimal surgical trauma, less blood loss, fewer hospital days, less pain, better presentation of the surgical area do to optical magnification, faster healing, and early recovery.^8^

So far, only three case reports with benign cystadenoma of the seminal vesicles have been described in the literature, which were effectively treated using DaVinci robot-assisted surgical resection.^9–11^

In the case of tumor recurrence, an open surgery with laparotomy *vs* minimally invasive laparoscopic surgery can be considered. After surgical removal of the tumor, a significant reduction or normalization of catecholamine release can be observed, reducing the need for an adrenergic blockade, daily medication intake, and a better response to the subsequent therapy.^2,12^

If left untreated, PGL may lead to elevated cardiovascular morbidity and mortality rate.^13^

In the primary tumor resection, a complete tumor removal should be performed. If possible, a careful and precise dissection should be carried out to prevent capsule rupture, as this may contribute to/predispose to late tumor recurrences in the surgical bed and seeding.^14^

Work-up of the histology specimen and diagnosing are difficult. There are no reliable criteria that indicate a malignant behavior. The definitive criterion of a malignant PGL, according to the WHO, is the development of metastases. In this case, a small size constant lung lesion was present for years.

In summary, most cases of PGL in the small pelvis are nonfunctional, surgical tumor excision is in most cases curative, and late recurrence is not common. Genetic testing for mutation was not routinely performed.

## Conclusion

In the event of paroxysmal severe headache with hypertensive derailments and vegetative symptoms, a catecholamine excess syndrome should be considered. The determination of metanephrine and normetanephrine levels in plasma and urine is a suitable screening test for catecholamine release in PGL. PGL are unusual in the small pelvis. Nevertheless, they should be included in the differential diagnosis of an incidentally found mass with features suggesting a vascular tumor. Possible symptoms may include LUTS, hematuria, headaches with an unknown cause, hematuria, hemospermia, hypertension or upper urinary tract obstruction. Including PGL in the differential diagnosis may facilitate biopsy diagnosis and guide further therapy in case of functionally dormant tumor before or during a surgery or a biopsy. An interdisciplinary approach with precise imaging to differentiate PHEO and PGL followed by sympathetic blockade and minimally invasive surgical tumor removal are recommended for therapy at centers with appropriate expertise to ensure a favorable outcome. Relevant for the clinical practice is a lifelong follow-up and regular examinations of patients to detect and prevent late recurrences or delayed appearance of multiple tumors.

## Consent

Written informed consent was obtained from the patient for publication of this case report. A copy of the written consent is available for review by the Editor-in-Chief of this journal.

## Abbreviations Used

CK: creatine kinase
CT: computed tomography
(18)F-DOPA-PET/CT: fluorodeoxyglucose-positron emission computed tomography
fT3: free triiodothyronine
LUTS: lower urinary tract symptoms
MIBG: metaiodobenzylguanidine scintigraphy
PGL: paraganglioma
PHEO: pheochromocytoma
PSA: prostate-specific antigen
TSH: thyroid-stimulating hormone
UUTO: upper urinary tract obstruction
